# Development of a short form of Mini-Mental State Examination for the screening of dementia in older adults with a memory complaint: a case control study

**DOI:** 10.1186/1471-2318-11-59

**Published:** 2011-10-04

**Authors:** Gladys Haubois, Cédric Annweiler, Cyrille Launay, Bruno Fantino, Laure de Decker, Gilles Allali, Olivier Beauchet

**Affiliations:** 1 Department of Internal Medicine and Geriatrics, Angers University Hospital, Angers, France; 2 Angers University Memory Clinic, Angers, France; 3 UPRES EA 2646, University of Angers, UNAM, France; 4 Department of Geriatrics, Nantes University Hospital, Nantes, France; 5 Department of Neurology, Geneva University Hospital and University of Geneva, Geneva, Switzerland

## Abstract

**Background:**

Primary care physicians need a brief and accurate screening test of dementia. The objective of this study was to determine whether a short form of Mini-Mental State Examination (SMMSE) was as accurate as the Mini-Mental State Examination (MMSE) in screening dementia.

**Methods:**

Based on case control design study, SMMSE and MMSE were assessed in 184 community-dwelling older adults (mean age 81.3 ± 6.5 years, 71.7% women) with memory complaint sent by their primary care physician to a memory clinic. Included participants were separated into two groups: cognitively healthy individuals and demented individuals.

**Results:**

The trade-off between sensitivity and specificity of the SMMSE for clinically diagnosed dementia was 4. Based on the cut-off value ≤ 4 for SMMSE and a cut-off value ≤ 24 for MMSE, the sensitivity of both tests was similar (89.5% for SMMSE versus 90.0% for MMSE), whereas the specificity, the positive predictive values (PPV) and the negative predictive values (NPV) were higher for SMMSE compared to MMSE (85.4 versus 75.5% for specificity; 95.5% versus 92.8% for PPV; 70.0 versus 68.9 for NPV). The positive and negative Likehood Ratio (LR) of SMMSE were higher than those of MMSE (respectively, 6.1 versus 3.7; 8.1 versus 7.7). In addition, odds ratio (OR) for dementia was higher for the SMMSE compared to the MMSE (OR = 49.8 with 95% confident interval (CI) [18.0; 137.8] versus OR = 28.6 with 95% CI [11.6; 70.3]).

**Conclusions:**

SMMSE seems to be an efficient short screening test for dementia among community-dwelling older adults with a memory complaint. Further research is needed to confirm its predictive values among unselected primary care older patients.

## Background

Dementia is an acquired clinical syndrome that associates memory decline and at least one other cognitive function decline, all disturbing social or daily living activities [1]. An estimated 50% of patients over 65 are not diagnosed by their primary care physician, and most missed cases are mild to moderate [2,3]. As yearly incidence of dementia increases with age, the under-diagnosis of dementia in primary care will probably increase in the future [3]. This under-diagnosis is in part related to a limited time for in-depth consideration of cognitive difficulties of primary care patients [1-3].

Since 2003, the U.S. preventive services task force highlighted that the Mini-Mental State Examination (MMSE) was the best-studied instrument to screen cognitive impairment in selected patients with cognitive impairment [1]. This test is scored out of 30, a score of 24 or less suggesting dementia [1-3]. Although the MMSE is the most widely used screening test in specialized clinical setting, it is little used by primary care physicians because it is time-consuming (e.g.; around 20 minutes to perform), and is thus hardly practicable during routine office visits [3]. Several short screening tests for dementia have been developed but their positive predictive values (PPV) were low, calculated around 50% [1]. Therefore, for dementia screening, primary care physicians need a brief and accurate screening test, applicable during clinical practice among patients with cognitive complaint.

Episodic memory is a memory system that enables individuals to remember events, times and places acquired through personal experience. Episodic memory impairment is the most frequent symptom of dementia and the most common complaint [1-3]. A brief cognitive test based on the evaluation of episodic memory impairment with sufficient sensitivity, specificity and predictive values could therefore serve as a useful screening tool for dementia. Among the 30 items of the MMSE that address multiple cognitive domains, 6 items specifically assessing episodic memory can be fortunately distinguished [4]. The time needed to complete these 6 episodic memory items is much shorter compared to the 30 items of the whole MMSE. What is more, since MMSE is widely used for a long time all over the world [1-3], physicians usually know it and the way to use it. These two points suggest that a short test based on the 6 episodic memory items of the MMSE would be easily used by physicians to detect dementia. Its accuracy remains yet to be determined. We hypothesized that the 6 memory items of the MMSE could be used alone to build a Short form of Mini-Mental State Examination (SMMSE) with sufficient sensitivity, specificity, positive and negative predictive values to serve as a good screening test for dementia in older adults with memory complaint. The aim of this study was to determine whether the SMMSE was as accurate as the MMSE in screening dementia among older ambulatory participants with a memory complaint.

## Methods

### Participants

On hundred eighty four participants (41 cognitively healthy individuals (CHI) and 143 demented individuals (DI)) were included in this case control design study between January 2009 and December 2009, after obtaining their informed consent or the agreement of the trusted person, as appropriate. Consent forms were acquired from all participants sent for a memory complaint by their primary care physician to the memory clinic of Angers University Hospital, France. The eligible criteria for inclusion in this study were age 65 and over, ambulatory, adequate ability to understand and speak French and no acute medical illness in the past month. The exclusion criteria were active depression and mild cognitive impairment. The classification of normal cognition and dementia were determined at a weekly interdisciplinary conference based on the neuropsychological and behavioural tests performance, as well as physical examination findings, blood values and brain imaging findings. The diagnosis of dementia followed the Diagnostic and statistical manual of mental disorders fourth edition (DSM-IV) criteria [4]. Alzheimer's disease (AD) and vascular dementia were diagnosed according to respectively the NINCDS-ADRDA criteria for probable AD and the criteria of the DSM-IV [5,6]. This study was in accordance with the ethical standards set forth in the declaration of Helsinki (1983). The local ethics committee of Angers University Hospital (Committee for the Protection of People West-2, Angers, France) approved the project.

### Neuropsychological evaluation

Neuropsychological evaluation was performed during a face-to-face examination carried out by a neuropsychologist. The following standardized tests were used to probe several aspects of cognitive function: the MMSE [4], the Cognitive Assessment Battery (CAB) [7], the Frontal Assessment Battery (FAB) [8], and the Instrumental Activities of Daily Living (IADL) [9]. The CAB assesses cognitive function as a whole (i.e., memory, language, praxia, spatiotemporal orientation and reasoning). It is composed of 96 items and its completion time is around 45 minutes. Score range varies from 0 to 96, with 96 indicating a normal cognitive functioning [7]. The FAB is a validated, short bedside questionnaire designed to assess both cognitive and behavioural changes in frontal lobe dysfunctions [8]. The FAB is a scale composed of 6 subtests (conceptualization, mental shifting, motor programming, sensitivity to interference, inhibitory control, and environment autonomy). It can be performed in approximately 10 minutes. Score range varies from 0 to 18, a score of 18 indicating normal executive functioning [8]. The IADL is a scale evaluating the autonomy to perform the instrumental activities of daily living (i.e., using transportation, managing finances, using the phone, managing medicines) [9]. Each affected function is marked 0. Score range varies from 0 to 4, a score under 3 indicating disability [9].

The SMMSE score was built from the 6 memory items of the MMSE. It was calculated following the formula: [immediate recall of 3 words + delayed recall of 3 words]. The score ranged from 0 to 6, with 6 indicating the best performance.

### Statistics

**Figure 1:**
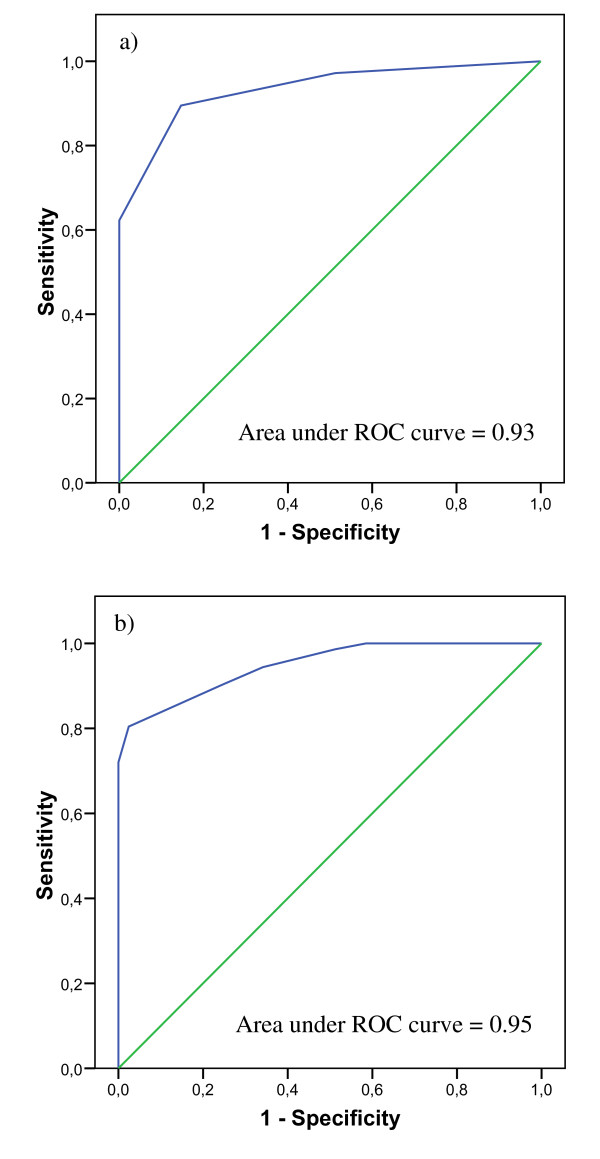
**Receiver operator characteristic curves for SMMSE and MMSE**. a) SMMSE. b) MMSE. SMMSE: Short form of Mini-Mental State Examination; MMSE: Mini-Mental State Examination; ROC: Receiver operator curve.

The participants' baseline characteristics were summarized using means and standard deviations or frequencies and percentages, as appropriate. Participants were separated into two groups: CHI and DI. Comparisons between groups were performed using t-test for independent samples or Chi-square, as appropriate. Uni and multiple logistic regression analyses were performed to specify the association between dementia (dependent variable) and SMMSE (independent variable) adjusted on baseline characteristics. Sensitivity analysis consisted of building a receiver operator curve (ROC) by computing the sensitivity and specificity of each SMMSE score (from 0 to 6), to determine the most discriminated threshold maximizing both sensitivity and specificity (Figure 1). Sensitivity, specificity, PPV, negative predictive value (NPV), positive and negative likehood ratio (LR), and odds ratio (OR) for dementia were calculated for the SMMSE based on the cut-off value determined by the sensitive analysis, and for the MMSE based on the cut-off value ≤ 24 [1-3]. P-values < 0.05 were considered as statistically significant. All statistics were performed using SPSS (version17.0; SPSS, Inc., Chicago, IL) and Dag-stat a spreadsheet for the calculation of comprehensive statistics for the assessment of diagnostic tests [10].

## Results

The diagnosis of dementia included 53.8% (n = 99) Alzheimer's disease (AD), 14.7% (n = 27) vascular dementia and 9.2% (n = 17) other dementia. DI were older than CHI (P < 0.001) and had lower score on MMSE (P < 0.001), SMMSE (P < 0.001), CAB (P < 0.001), FAB (P < 0.001) and IADL (P < 0.001) (Table 1). There was no significant between-group difference regarding the other clinical characteristics. The ROC curve showed an area of 0.93 for SMME and of 0.95 for MMSE (Figures 1a and 1b). In logistic regression models, SMMSE score was negatively associated with dementia (P < 0.001) and age was positively associated with dementia (P < 0.001 for unadjusted model, P = 0.002 for fully adjusted) (Table 2). The trade-off between sensitivity and specificity of the SMMSE for clinically diagnosed dementia was 4. Based on the cut-off value ≤ 4 for SMMSE and a cut-off value ≤ 24 for MMSE, the sensitivity of both tests was similar (89.5% for SMMSE versus 90.0% for MMSE), whereas the specificity, the PPV and the NPV were higher for SMMSE compared to MMSE (respectively, 85.4 versus 75.5% for specificity; 95.5% versus 92.8% for PPV; 70.0 versus 68.9 for NPV). The positive and negative LR of SMMSE were higher than those of the MMSE (respectively, 6.1 versus 3.7; 8.1 versus 7.7). In addition, OR for dementia was higher for the SMMSE than the MMSE (OR = 49.8 with 95% confident interval (CI) [18.0;137.8] versus OR = 28.6 with 95% CI [11.6;70.3]) (Table 3).

**Table 1:** Clinical characteristics of studied sample of participants according to diagnostic of dementia (n = 184)

	CHI (n = 41)	DI (n = 143)	P-value*
Age, mean ± SD (years)	77.7 ± 7.7	82.3 ± 5.7	**< 0.001**
Female, n (%)	30 (73.2)	102 (71.3)	1.000
Use of psychoactive drugs daily†, n (%)	14 (34.1)	70 (49.0)	0.111
MMSE score (/30 points), mean ± SD	26.7 ± 2.2	19.4 ± 4.1	**< 0.001**
SMMSE score (/6 points), mean ± SD	5.3 ± 0.7	3.4 ± 0.9	**< 0.001**
CAB score (/96 points), mean ± SD	84.8 ± 6.3	67.2 ± 11.3	**< 0.001**
FAB score (/18 points), mean ± SD	14.2 ± 3.2	10.7 ± 2.8	**< 0.001**
IADL score (/4 points), mean ± SD	3.9 ± 0.3	2.4 ± 0.7	**< 0.001**

CHI: Cognitively healthy individuals; DI: demented individual; MMSE: Mini-Mental State Examination; SMMSE: Short form of Mini-Mental State Examination (maximum score of 6 corresponding to best performance); CAB: Cognitive Assessment Battery; FAB: Frontal Assessment Battery; IADL: Instrumental Activities of Daily Living; *: Comparison based on t-test or Chi-square test, as appropriate; † Use of benzodiazepines or antidepressants or neuroleptics

**Table 2:** Uni and multivariate logistic regression models showing the association between dementia (dependent variable) and SMMSE score (independent variable) and adjusted on clinical characteristics (n = 184)

	Unadjusted model			Fully adjusted model		
	
	OR	95% CI	P-value	OR	95% CI	P-value
SMMSE	**0.12**	[0.07; 0.22]	**< 0.001**	**0.10**	[0.05; 0.21]	**< 0.001**
Age	**1.12**	[1.06;1.19]	**< 0.001**	**1.17**	[1.06;1.29]	**0.002**
Female	0.91	[0.42;1.99]	0.817	1.29	[0.38;4.42]	0.688
Psychoactive drugs†	1.85	[0.90;3.81]	0.096	1.01	[0.33;3.15]	0.986

SMMSE: Short form of the Mini-Mental State Examination (maximum score of 6 corresponding to best performance); OR: odds ratio; *: Use of benzodiazepines or antidepressants or neuroleptics; OR and P-value significant (i.e.; P-value < 0.05) indicated in bold.

**Table 3:** Performance of SMMSE and MMSE for the diagnosis of dementia (n = 184)

	SMMSE score ≤ 4	MMSE score ≤ 24
Sensitivity (%)	89.5	90.0
Specificity (%)	85.4	75.5
Positive predictive value (%)	95.5	92.8
Negative predictive value (%)	70.0	68.9
Likelihood ratio of positive test	6.1	3.7
Likelihood ratio of negative test	8.1	7.7
Odds ratio for dementia [95% CI]	49.8 [18.0;137.8]	28.6 [11.6;70.3]

SMMSE: Short form of Mini-Mental State Examination

MMSE: Mini-Mental State Examination

CI: Confidence interval

## Discussion

Our results showed that the SMMSE score was strongly associated with the diagnosis of dementia. In addition, a score ≤ 4 of SMMSE had a higher specificity, PPV, NPV, positive and negative LR than a MMSE score ≤ 24.

The SMMSE appears to be a good new screening test for dementia among older adults presenting with a memory complaint. We showed a strong association between a score ≤ 4 and dementia which is in concordance with previous studies reporting that memory tests had a high discriminative validity for dementia [1-3,11]. AD has been identified as the most prevalent dementia in older adults, which was the case in our studied sample [1]. Because memory impairments are highly prevalent in AD, it is not surprising that a test based on memory may screen dementia with a high predictive value [1,11]. However, compared to previous studies, SMMSE did not use an encoding specificity because it follows the instructions of assessment of MMSE [1-3,11]. SMMSE thus assesses not only episodic memory (encoding and retrieval phase) but also semantic memory, attention and probably executive functions deficit.

In clinical practice, when older patients present with a memory complaint, the first issue is to make a diagnosis and determine whether they are demented or not. A screening test able to identify patient who have dementia is therefore of prime importance for daily practice [1,11]. Discriminative validity of SMMSE for dementia was high compared to MMSE in our study. In addition, unlike previous short screening tests for dementia, SMMSE had a higher PPV than MMSE calculated at 95.5 [1]. The SMMSE could thus be used in clinical practice as an efficient screening test for dementia in older adults with a memory complaint.

The second issue in primary care settings is related to the under-diagnosis of dementia [1-3]. Several explanations account for this problem which is not only due to the lack of diagnostic skill, but rather to a pressure on time [3]. Because MMSE takes up to 20 minutes to complete, it may not be practical in primary care practice. In contrast, SMMSE is shorter to perform, easier and more accessible compared to MMSE. As a consequence, SMMSE could be proposed to general practitioners as a fast screening tool for dementia.

Our findings showed that DI were older than CHI and that age was significantly associated with dementia. This was in concordance with previous studies as it is long known that the primary risk factor of dementia is the advance in age [6,7,11]. We also reported that there were no association between the female gender or the use of psychoactive drugs and the diagnosis of dementia. These results were discordant with some previous published data having identified female gender as a risk factor of dementia [12]. Furthermore, demented patients frequently use psychoactive drugs due to behavioral disorders [13]. An explanation for our mixed results could rely on the fact that both groups of participants were old in our study, with a proportion of females using psychoactive drugs that is usually high among this population [14]. This phenomenon could mask the classical associations with gender and psychoactive drugs in our sample of demented patients.

Our study has several limitations. First, we used of a case control design which may limit the exploration of the association between SMMSE score and dementia, compared to a cohort design. Second, although we were able to control for many confounders likely to modify the relationship between SMMSE score and dementia, residual potential confounders may still be present. Third, SMMSE was built from MMSE score and not performed as a single test that may probably induce variation in score.

## Conclusions

SMMSE seems to be a short screening test for dementia in older adults with memory complaint. Further research is needed to confirm its predictive values in primary care older patients.

## List of abbreviations

SMMSE: Short form of Mini-Mental State Examination; MMSE: Mini-Mental State Examination; PPV: Positive predictive values, NPV: Negative predictive values, CHI: Cognitively healthy individuals, DI: Demented individuals; DSM-IV: Diagnostic and statistical manual of mental disorders fourth edition; AD: Alzheimer's disease; FAB: Frontal Assessment Battery; IADL: Instrumental activity daily living; LR: Likehood ratio; OR: Odds ratio.

## Competing interests

The authors declare that they have no competing interests.

## Authors' contributions

Study concept and design (OB and BF); acquisition of data (GH); analysis and interpretation of data (OB, CA, LD, CL, GA); Drafting of the manuscript (OB, BF, GH); critical revision of the manuscript for important intellectual content (CA, LD, CL, GA); obtained funding (Not applicable); statistical expertise (OB); administrative, technical, or material support (GH); Study supervision (BF and OB).

All authors (i.e.; GH, CA, BF, LD, GA, OB) read and approved the final manuscript. GH has full access to the data in the study and takes responsibility for the integrity of the data and the accuracy of the data analyses.

## Source(s) of Funding

None

## Previous presentations of the whole or part of the work presented in the article

None

## Pre-publication history

The pre-publication history for this paper can be accessed here:

http://www.biomedcentral.com/1471-2318/11/59/prepub
